# The integrity of dopaminergic and noradrenergic brain regions is associated with different aspects of late-life memory performance

**DOI:** 10.1038/s43587-023-00469-z

**Published:** 2023-08-31

**Authors:** Martin J. Dahl, Shelby L. Bachman, Shubir Dutt, Sandra Düzel, Nils C. Bodammer, Ulman Lindenberger, Simone Kühn, Markus Werkle-Bergner, Mara Mather

**Affiliations:** 1 Center for Lifespan Psychology, Max Planck Institute for Human Development, Berlin, Germany; 2 Leonard Davis School of Gerontology, University of Southern California, Los Angeles, CA USA; 3 Department of Psychology, University of Southern California, Los Angeles, CA USA; 4 Max Planck UCL Centre for Computational Psychiatry and Ageing Research, London, UK; 5 Max Planck UCL Centre for Computational Psychiatry and Ageing Research, Berlin, Germany; 6 Lise Meitner Group for Environmental Neuroscience, Max Planck Institute for Human Development, Berlin, Germany; 7 Department of Psychiatry and Psychotherapy, University Clinic Hamburg-Eppendorf, Hamburg, Germany; 8 Department of Biomedical Engineering, University of Southern California, Los Angeles, CA USA

**Keywords:** Cognitive ageing, Predictive markers, Long-term memory, Ageing

## Abstract

Changes in dopaminergic neuromodulation play a key role in adult memory decline. Recent research has also implicated noradrenaline in shaping late-life memory. However, it is unclear whether these two neuromodulators have distinct roles in age-related cognitive changes. Here, combining longitudinal MRI of the dopaminergic substantia nigra–ventral tegmental area (SN-VTA) and noradrenergic locus coeruleus (LC) in younger (*n* = 69) and older (*n* = 251) adults, we found that dopaminergic and noradrenergic integrity are differentially associated with memory performance. While LC integrity was related to better episodic memory across several tasks, SN-VTA integrity was linked to working memory. Longitudinally, we found that older age was associated with more negative change in SN-VTA and LC integrity. Notably, changes in LC integrity reliably predicted future episodic memory. These differential associations of dopaminergic and noradrenergic nuclei with late-life cognitive decline have potential clinical utility, given their degeneration in several age-associated diseases.

## Abstract (web summary)

Using multimodal MRI to delineate noradrenergic and dopaminergic nuclei in aging, Dahl et al. found differential associations with episodic and working memory, helping to disentangle the role of the neurotransmitter systems in age-related memory loss.

## Main

Our memory fades as we age^1^. On average, old age is characterized by impaired abilities to retain and manipulate information over short timescales—termed working memory^2,3^—and to recollect past experiences with their temporal and spatial context—called episodic memory^1,4,5^. On a neural level, senescent declines in memory have been linked to dopaminergic neuromodulation^6,7^ and, more recently, also to noradrenergic neuromodulation^8–10^. The degeneration of catecholaminergic (that is, dopaminergic and noradrenergic) systems also is a core feature of age-related pathologies, such as Alzheimer’s disease and Parkinson’s disease^9,11–15^, which are characterized by amnestic impairments^16,17^. However, investigations disentangling the contribution of the two neuromodulators to human memory in aging and disease are scarce.

Neuromodulators are neurochemicals synthesized in circumscribed subcortical nuclei. Widely branching axonal projections from these nuclei release these neuromodulators throughout the brain^18^. Dopaminergic neurons are based mainly in the midbrain substantia nigra–ventral tegmental area (SN-VTA)^19^, whereas noradrenergic neurons are primarily found in the brainstem locus coeruleus (LC)^20^.

Several mechanistic accounts link dopaminergic and noradrenergic neuromodulation to aging memory. Computational models posit that catecholamines modulate the input–output relation of neurons (that is, gain change), which increases the signal-to-noise ratio in neural processing^21^ and influences cognition^22–24^. Age-related neurodegeneration of dopaminergic and noradrenergic nuclei thus results in noisier neural information processing (that is, gain reduction)^7^. Specifically, declining catecholaminergic drive with increasing age is hypothesized to lead to less distinctive cortical representations and senescent memory decline^7,25^.

A second mechanism linking dopaminergic and noradrenergic neuromodulation to aging memory is their modulation of prefrontal processing^26^. Lateral prefrontal circuits can represent external stimuli in the absence of sensory stimulation, even in the face of distractors, by means of persistently firing delay cells^27^. Catecholaminergic inputs orchestrate recurrent activity in delay cell circuits that is essential for higher-order cognitive functions, such as working memory^28^. Specifically, the stimulation of dopaminergic D1-receptors and noradrenergic α_2a_-receptors boosts prefrontal delay activity with an inverted-u dose–response curve^10^. Age-related memory deficits, in turn, have been associated with reduced delay cell firing, which could be partially restored by catecholaminergic drugs^29–31^.

Finally, dopamine and noradrenaline modulate hippocampal long-term potentiation and long-term depression^32–36^, which are critical for synaptic plasticity and memory. Initial accounts proposed a ventral tegmental area–hippocampal circuit by which neuromodulatory inputs facilitate the consolidation of salient experiences^32,35^. Interestingly, more recent investigations indicate that, although the SN-VTA and LC both project to the dorsal hippocampus, the latter sends denser inputs^37–39^. LC neurons also produce dopamine as biosynthetic precursor of noradrenaline and can co-release both catecholamines to modulate hippocampal synaptic plasticity and memory^38,40^. Older age is characterized by impaired hippocampal plasticity^41,42^, likely exacerbated by deficient catecholaminergic innervation from the LC and SN-VTA^43^.

Taken together, dopaminergic and noradrenergic neuromodulation mechanistically sculpts senescent memory via several pathways, including gain modulation^7,8^, frontal delay activity^27,29^ and hippocampal synaptic plasticity^32,40^. Notably, these mechanisms are specified for dopaminergic and noradrenergic neuromodulation (gain modulation^21^, delay activity^10^ and synaptic plasticity^40^). However, research sampling a broad array of cognitive tasks to identify unique associations with late-life memory is lacking. That is, although animal research has demonstrated considerable overlap of catecholamines at the neural level, the question remains how much dopaminergic and noradrenergic nuclei overlap in their association with behavior.

Comparative studies of catecholaminergic systems in humans were long hampered by technical challenges in reliable non-invasive assessments of the small subcortical nuclei^44,45^. However, recent advances in high-resolution magnetic resonance imaging (MRI) reveal the SN-VTA^14,46^ and LC^47,48^ as focal hyperintensity on MRI scans. Multimodal postmortem validation studies suggest this hyperintensity as a marker for catecholaminergic neurons^49–51^. Neuromelanin, a catecholamine-derived paramagnetic pigment accumulating in the LC and SN-VTA^52,53^, presumably contributes to the MRI contrast of the nuclei^48,50^. However, other factors also likely play a role, such as the large cellular bodies of catecholaminergic neurons^54,55^ that result in a high abundance of ions and water protons^55^ as well as a lower macromolecular fraction^56,57^. Importantly, first in vivo studies suggest an association between the MRI intensity of catecholaminergic nuclei and their functionality^50^. Furthermore, investigations in clinical populations confirm the validity of the imaging approach^14,58–62^.

Catecholaminergic nuclei are among the first brain structures to accumulate pathologies in age-associative diseases, such as Parkinson’s and Alzheimer’s^11,63,64^, and show severe degeneration over the course of these diseases^9,13,65^. In line with this, LC^58–60^ and SN-VTA^14,61,62^ imaging using dedicated MRI sequences (that is, Magnetization Transfer (MT) and Fast Spin Echo (FSE)) reveals reduced hyperintensities in patients relative to controls. In healthy lifespan samples, initial cross-sectional evidence reveals a negative quadratic relationship between age and catecholaminergic hyperintensity^66–68^, whereby lower contrast with advancing age might be linked to impending pathology^58,69,70^. Taken together, recent advances in imaging techniques open the door for comparative non-invasive assessments of catecholaminergic nuclei integrity, which are sensitive for age-related and disease-related changes^14,46,48,61,62^.

In the present study, we took advantage of these new imaging approaches to attempt to disentangle the relative contribution of declining dopaminergic and noradrenergic neuromodulation to aging cognition. We repeatedly assessed cognitive performance and high-resolution MRI in large samples of younger and older adults across multiple timepoints^71–74^. Furthermore, we leveraged latent-variable modeling of multimodal imaging^75,76^ and comprehensive cognitive assessments^76,77^ to decrease measurement error and increase generalizability^78^. In sum, the goal of this study was to extend knowledge about the respective roles of dopaminergic and noradrenergic neuromodulation in late-life memory decline.

## Results

### LC and SN-VTA intensity shows high agreement across modalities

**Fig. 1:**
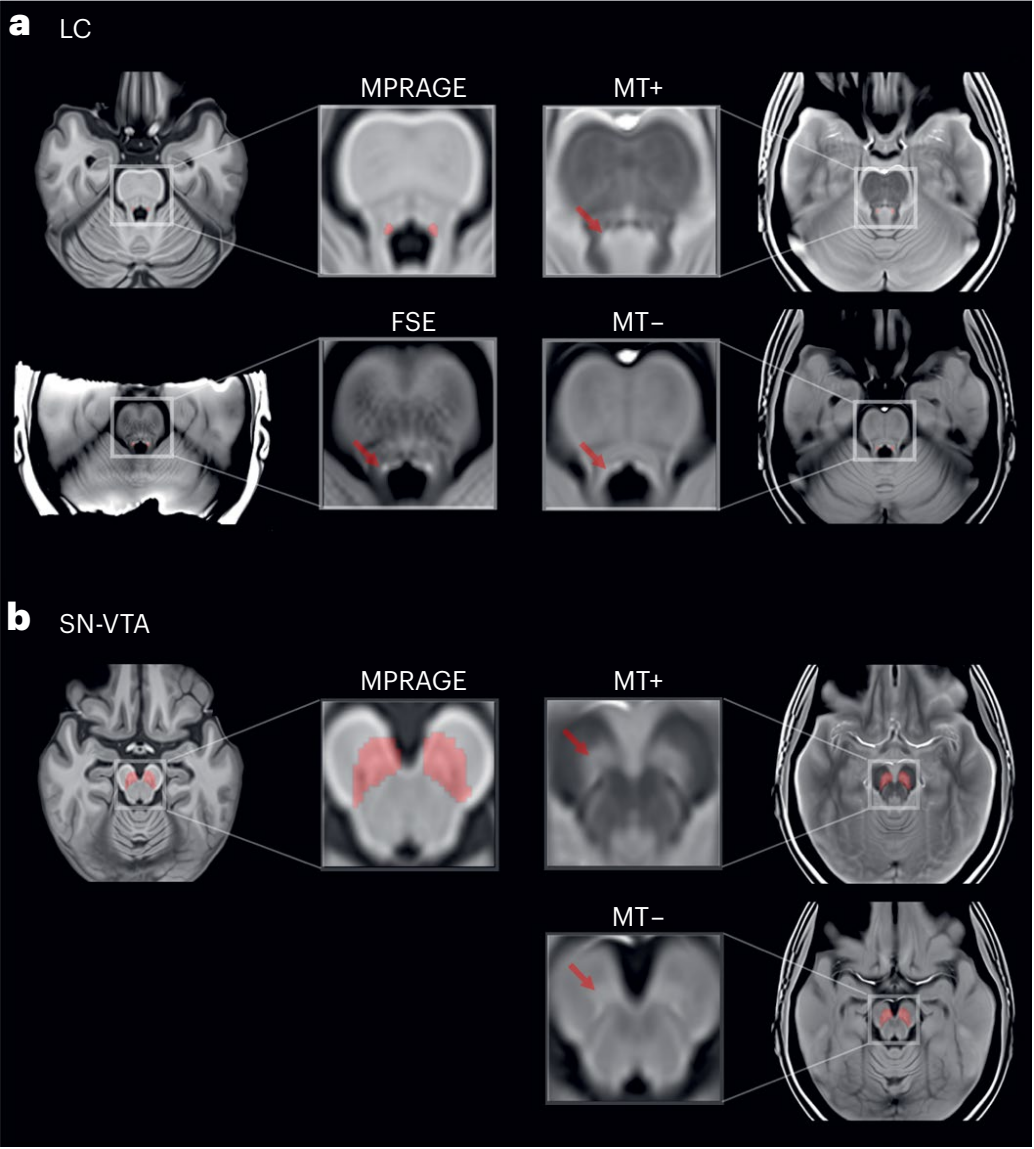
LC and SN-VTA-sensitive MRI sequences. **a**,**b**, Hyperintensities corresponding to the LC (**a**) and SN-VTA (**b**) are indicated by red arrows on axial slices of group templates based on an FSE sequence, and an MT sequence, acquired once with a dedicated magnetic saturation pulse (MT+) and once without, resulting in a proton density image (MT−). An MPRAGE sequence template shows the location of a previously established LC^58^ and SN-VTA^50^ volume of interest (red overlays). Note that our FSE sequence covered only the brainstem. All templates were registered to MNI152 0.5-mm linear space and are available in ref. ^84^. Templates were estimated across age groups.

**Fig. 2:**
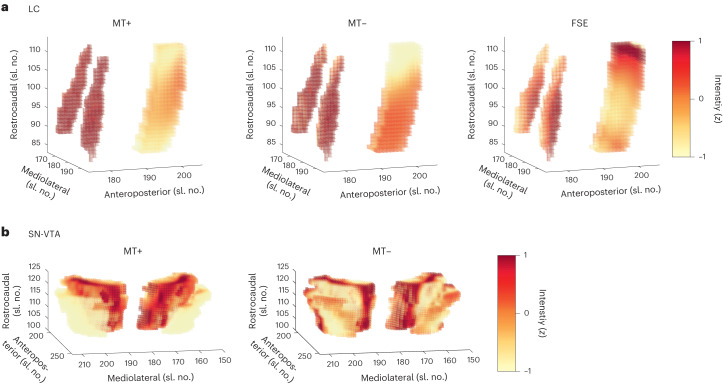
Normalized intensity in the LC, SN-VTA and corresponding white matter reference regions. **a**,**b**, Hyperintensities corresponding to the LC (**a**) and SN-VTA (**b**) are evident in group templates based on an FSE sequence, and a MT sequence, acquired once with a dedicated magnetic saturation pulse (MT+) and once without, resulting in a proton density image (MT−). LC and reference volumes of interest are taken from ref. ^58^. SN-VTA and reference volumes of interest are based on ref. ^50^. The reference regions are located anterior of the LC and SN-VTA, respectively. In the visualization, they can be seen as rectangular shapes to the right of the LC (that is, the two red columns) as well as in front of the SN-VTA (that is, the red curved shape). Note that our FSE sequence covered only the brainstem. Templates were estimated across age groups. sl. no., slice number.

We applied a validated semiautomatic procedure^58,79–82^ to extract intensity information of catecholaminergic nuclei from different imaging sequences (FSE, MT+ and MT−; Methods and Supplementary Fig. 2) by standardizing MRI intensity in the LC and SN-VTA based on the intensity in an adjacent white matter reference region^47,48,50^ (Figs. 1 and 2). Based on earlier postmortem validations^49–51,59^, we interpret individual differences in standardized MRI intensity as proxy for the integrity of catecholaminergic nuclei. Next, we leveraged an established factor structure^79^ to integrate intensity information across hemispheres for each imaging sequence and age group (Supplementary Fig. 3).

**Fig. 3:**
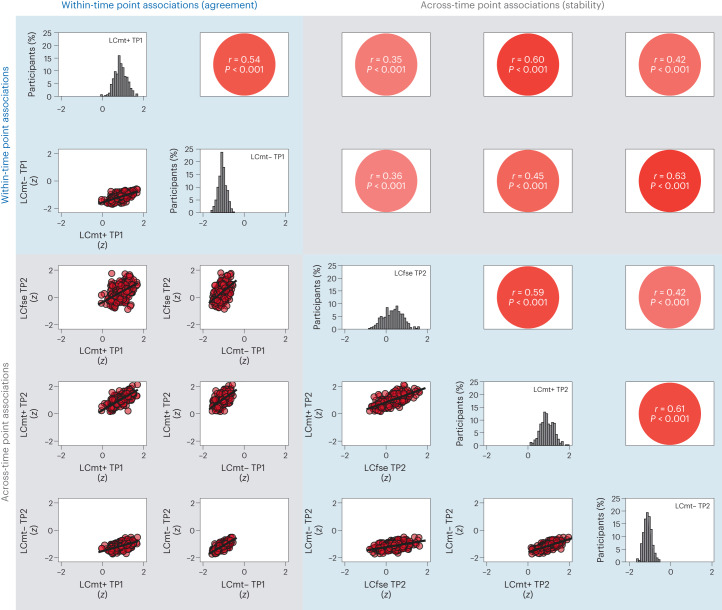
Younger and older adult LC intensities are correlated across imaging modalities—a marker for their agreement—and across timepoints—a marker for their stability. Visualized data are based on the statistical model depicted in Supplementary Fig. 14. For the same analyses using SN-VTA data, see Supplementary Figs. 18 and 20. Note, the diagonal shows LC intensity, standardized across all sequences and timepoints, to facilitate comparing intensity distributions. Imaging sequences included an FSE sequence, and an MT sequence, acquired once with a dedicated magnetic saturation pulse (MT+) and once without, yielding a proton density image (MT−). *n* = 320 biologically independent participants. Statistics are based on two-sided likelihood-ratio tests without additional adjustment for multiple comparisons. For full test statistics, see Supplementary Table 3.

Previous in vivo studies of catecholaminergic nuclei relied on different imaging approaches (mostly MT+ and FSE^14,46–48^), but there are few comparisons between these MRI sequences, limiting cross-study comparability. Contrasting LC and SN-VTA estimates, we found strong differences across MRI sequences in their average intensity, Δ*χ*²(*df* = 2) = 693.55; *P* < 0.001 for older adult LC; Δ*χ*²(*df* = 1) = 657.37; *P* < 0.001 for older adult SN-VTA. That is, standardized to a reference region, catecholaminergic nuclei appeared brightest in the MT+, followed by the FSE and, finally, the MT− sequences, for older adult LC (mean (s.e.)): MT+, 25.816 (0.304); FSE, 20.144 (0.37); MT−, 6.425 (0.194); for older adult SN-VTA: MT+, 19.523 (0.235); MT−, 2.979 (0.135). Crucially, despite these mean differences, intensities were highly correlated across imaging modalities, *r* = 0.43–0.621; *P* < 0.001 for older adult LC; *r* = 0.503; *P* < 0.001 for older adult SN-VTA (Fig. 3 and Supplementary Figs. 6–8), indicating that these sequences provide convergent information about the same underlying construct (that is, catecholaminergic nuclei).

**Fig. 4:**
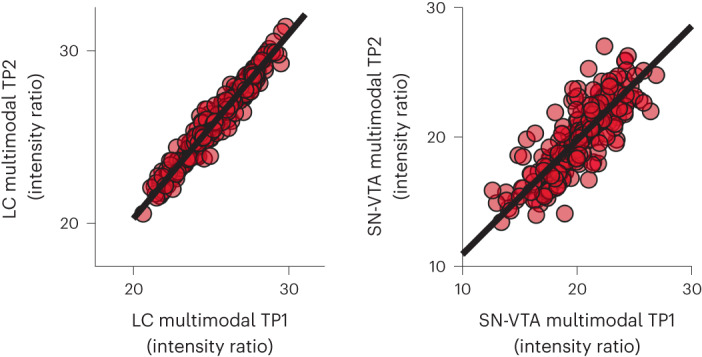
High stability of multimodal LC and SN-VTA factors. Multimodal LC and SN-VTA estimates show high stability over timepoints (mean delay ~1.9 years), *r* = 0.88; *P* < 0.001 for younger and older adult LC (test against mean modality-specific stability—that is, *r* = 0.615, *z* = 11.713; *P* < 0.001); *r* = 0.67; *P* < 0.001 for younger and older adult SN-VTA (test against mean modality-specific stability—that is, *r* = 0.448, *z* = 5.837; *P* < 0.001). *n* = 320 biologically independent participants. Statistics are based on two-sided likelihood-ratio tests without additional adjustment for multiple comparisons. For full test statistics, see Supplementary Table 3.

We thus aggregated the information shared across imaging modalities by estimating multimodal latent factors expressing LC integrity and SN-VTA integrity (Supplementary Fig. 4; for similar approaches and a discussion, see refs. ^75,76,83^). Such latent variables capture the commonalities across scan modalities while removing the modality-specific measurement error and, hence, increase statistical power to detect true effects^78^. Model visualizations, model fit and younger adult findings are reported in Supplementary Information (Supplementary Figs. 3, 4, 6, 7 and ref. ^84^).

Taken together, we extracted the intensity of the LC and SN-VTA from different MRI sequences sensitive for catecholaminergic nuclei. We found a high agreement in intensities across imaging modalities and, thus, aggregated across sequences to obtain individual integrity estimates for the two catecholaminergic nuclei.

### LC and SN-VTA integrity show high stability over time

This dataset’s longitudinal nature allowed us to examine the stability of our integrity estimates over time (TP1–TP2; mean delay ~1.9 (s.d. 0.7) years), as a proxy for their reliability^85^. Longitudinal studies investigating the reliability of imaging sequences for catecholaminergic nuclei are sparse. Thus, as a reference, we first assessed the stability of the modality-specific intensity factors and found evidence for an intermediate stability, for younger and older adult LC: MT+, *r* = 0.6; *P* < 0.001; MT−, *r* = 0.63; *P* < 0.001; for younger and older adult SN-VTA: MT+, *r* = 0.66; *P* < 0.001; MT−, *r* = 0.18; *P* = 0.17 (Fig. 3 and Supplementary Figs. 14, 18 and 20; FSE sequence only available for TP2 (Methods)). For similar analyses using intensity values extracted from the pontine reference (LC) and crus cerebri reference (SN-VTA) regions, see Supplementary Figs. 23 and 24. If our multimodal integrity factors remove modality-specific measurement error^76^, we should observe a higher stability across timepoints for the multimodal as compared to the modality-specific factors. Indeed, our multimodal LC and SN-VTA factors evinced a higher stability, pointing to the benefits of the multimodal imaging approach, *r* = 0.88; *P* < 0.001 for younger and older adult LC (test against mean modality-specific stability—that is, *r* = 0.615, *z* = 11.713; *P* < 0.001); *r* = 0.67; *P* < 0.001 for younger and older adult SN-VTA (test against mean modality-specific stability—that is, *r* = 0.448, *z* = 5.837; *P* < 0.001)^86^ (Fig. 4).

### LC and SN-VTA are related to different aspects of memory

**Fig. 5:**
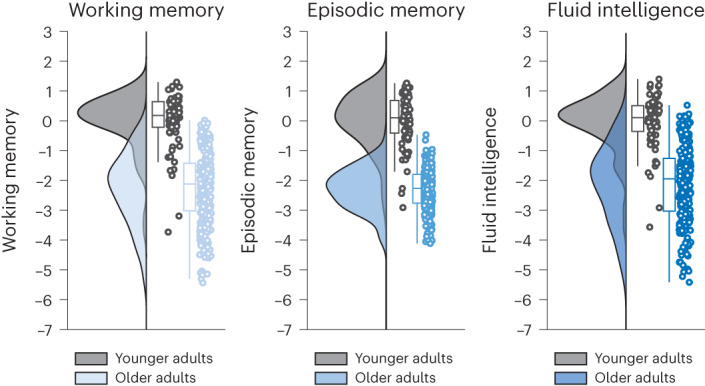
Cross-sectional age differences in working memory, episodic memory and fluid intelligence factors at TP2. Older adults show lower performance relative to younger adults across cognitive domains (mean age difference (s.e.)): working memory, −2.265 (0.309); episodic memory, −2.287 (0.309); fluid intelligence, −2.073 (0.295); all *P* < 0.001. Raincloud plots are based on ref. ^146^. For visualizations of episodic memory and working memory performance over TP1–3, see Supplementary Figs. 27 and 28. *n* = 320 biologically independent participants. Statistics are based on two-sided likelihood-ratio tests without additional adjustment for multiple comparisons. For full test statistics, see Supplementary Table 3. Box plots are defined by the following values: lower and upper bounds of the box, quartiles (0.25 (Q1) and 0.75 (Q3)); center of the box, quartile 0.5 (Q2); lower whisker (Q1 − 1.5 × interquartile range); upper whisker (Q3 + 1.5 × interquartile range). For the statistical model, see Supplementary Fig. 9.

Next, we cross-sectionally probed the behavioral implications of inter-individual differences in LC and SN-VTA integrity, using data of TP2. For this, we leveraged a comprehensive cognitive battery and a previously established factor structure^77^ to aggregate performance across several working memory, episodic memory and fluid intelligence tasks and capture their shared variance on a latent level (Supplementary Fig. 9). We observed strong age differences in each of the cognitive domains (Fig. 5), older relative to younger adults (mean age difference (s.e.)): working memory, −2.265 (0.309); episodic memory, −2.287 (0.309); fluid intelligence, −2.073 (0.295); all *P* < 0.001. Notably, however, when we added estimates for the catecholaminergic nuclei to the model, we found that higher LC and SN-VTA integrity were related to better performance (that is, less age-related cognitive impairments) across domains, Δ*χ*²(*df* = 3) = 25.11; *P* < 0.001 for older adult LC; Δ*χ*²(*df* = 3) = 7.86; *P* = 0.049 for older adult SN-VTA (Supplementary Fig. 10).

**Fig. 6:**
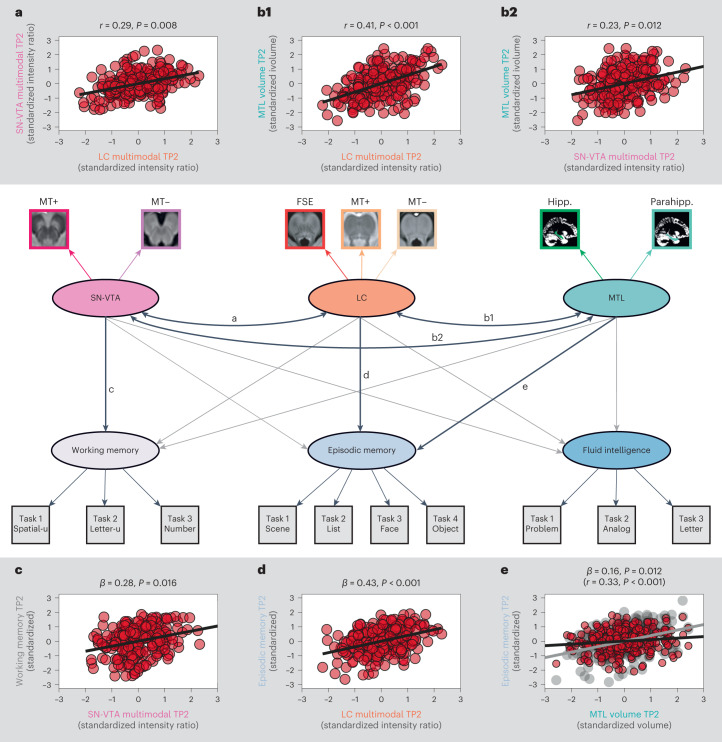
Cross-sectional associations of LC and SN-VTA factors with late-life cognition at TP2. Schematic pictorial rendition of a structural equation model probing the interrelation of catecholaminergic nuclei and medial temporal lobe volume (paths **a** and **b**) and their unique associations with late-life cognition (paths **c**–**e**) in older adults at TP2. Note that covariances among cognitive factors, intercepts and error terms are omitted for clarity. For the full statistical model, see Supplementary Fig. 13. Task*, cognitive paradigm (indicator; (square)) for the respective cognitive domain (latent factor; (ellipse)); one-headed arrows, regression; double-headed arrow, correlation. Note that the brightness of paths indicates their significance. Medial temporal lobe volumes demonstrate a reliable association only with episodic memory in a correlational model (gray line in scatter plot **e**; Supplementary Fig. 12) but not when controlling for catecholaminergic neuromodulation (black line in scatter plot **e**). LC and SN-VTA factors are derived from an FSE sequence, and an MT sequence, acquired once with a dedicated magnetic saturation pulse (MT+) and once without, resulting in a proton density image (MT−). Hipp, hippocampus volume; Parahipp, parahippocampus volume. *n* = 251 biologically independent participants. Statistics are based on two-sided likelihood-ratio tests without additional adjustment for multiple comparisons. For full test statistics, see Supplementary Table 3.

Dopaminergic and noradrenergic neuromodulatory centers are densely interconnected^34,87^. In addition, dopamine is the biosynthetic precursor of noradrenaline^88^, and, indeed, we detected a positive association between the structural metrics of the two neuromodulatory nuclei, *r* = 0.25; Δχ²(*df* = 1) = 5.75; *P* = 0.017 for older adults (compare to Fig. 6 and Supplementary Fig. 10). However, although the LC and SN-VTA were positively coupled, they differed in their association with late-life cognition, Δχ²(*df* = 3) = 15.66; *P* = 0.001 for older adults.

Follow-up analyses showed that higher LC integrity was related to better episodic memory performance, *r* = 0.49; Δχ²(*df* = 1) = 21.44; *P* < 0.001 for older adults (compare to Fig. 6 and Supplementary Fig. 10) and that this association was specific: that is, LC’s relation to cognition differed across domains (episodic memory, working memory and fluid intelligence), Δχ²(*df* = 2) = 10.64; *P* = 0.005 for older adults. Moreover, the LC–episodic memory association was stronger than that of the SN-VTA and episodic memory, Δχ²(*df* = 1) = 6.63; *P* = 0.01 for older adults. Taken together, our findings show a general (that is, task and imaging sequence independent) yet specific relation of LC and late-life episodic memory performance, corroborating and extending earlier work^58,59,79,89,90^.

In contrast to the LC, higher SN-VTA integrity correlated with better late-life working memory, *r* = 0.28; Δχ²(*df* = 1) = 6.76; *P* = 0.009 for older adults (compare to Fig. 6 and Supplementary Fig. 10). There was a numerical tendency for differential associations of SN-VTA integrity with performance in each of the cognitive domains (working memory, episodic memory and fluid intelligence), Δχ²(*df* = 2) = 5.73; *P* = 0.057 for older adults. However, we did not observe reliable differences in the relation of the two neuromodulatory nuclei to working memory, Δχ²(*df* = 1) = 2.01; *P* = 0.156 for older adults. In sum, our findings suggest a differential association of the two neuromodulatory systems with late-life memory performance. While episodic memory was associated with LC integrity, SN-VTA integrity was related to working memory.

Note that, in our model (Supplementary Fig. 10), correlations between the latent catecholaminergic and cognitive factors were computed for one neuromodulatory system without considering variance accounted for by the other neuromodulatory system. Thus, we additionally specified a second, statistically equivalent model in which we searched for unique associations with cognition for each catecholaminergic system while controlling for the respective other system, using a multiple regression approach (see ref. ^76^ for a similar approach; Fig. 6 and Supplementary Fig. 11). Crucially, we again detected reliable LC–episodic memory, *β* = 0.5; Δχ²(*df* = 1) = 19.55; *P* < 0.001 and SN-VTA–working memory associations, *β* = 0.28; Δχ²(*df* = 1) = 6.05; *P* = 0.014, for older adults, underlining differential relations to cognition of the two neuromodulatory nuclei. Younger and older adults showed similar associations between neuromodulatory integrity and memory performance (test for age group differences in (1) the LC–episodic memory association: (Δ*χ*²(*df* = 1) = 0.43; *P* = 0.512; and (2) the SN-VTA–working memory association: Δ*χ*²(*df* = 1) = 0.28; *P* = 0.594)). Model visualizations, model fit and younger adult findings are reported in Supplementary Information (Supplementary Figs. 10 and 11 and ref. ^84^).

### LC and SN-VTA are related to memory beyond medial temporal lobe volumes

Via direct projections, the LC and SN-VTA release catecholamines in memory-relevant brain regions, such as the medial temporal lobe (MTL)^37,39,40^, which improves retention performance^32,34,37,39,40,91^. Abnormally phosphorylated tau, an indicator of neurodegenerative diseases such as Alzheimer’s, starts to accumulate early in life in catecholaminergic nuclei^11,92–94^. With advancing age, abnormal tau also appears in projection targets of the neuromodulatory nuclei, such as the MTL^11,93,95,96^, which may facilitate degeneration^58,59,96,97^. Thus, as a control analysis, we also incorporated MTL volumes in our models linking catecholaminergic nuclei and cognition (Supplementary Figs. 12 and 13).

In a correlational model, we observed that the integrity of both catecholaminergic nuclei was positively associated with MTL volumes, *r* = 0.41; Δχ²(*df* = 1) = 27.45; *P* < 0.001 for older adult LC; *r* = 0.23; Δχ²(*df* = 1) = 6.29; *P* = 0.012 for older adult SN-VTA (Fig. 6 and Supplementary Fig. 12), potentially indicating neuroprotective catecholaminergic effects^98,99^. In addition, higher MTL volumes were related to better late-life episodic memory performance, *r* = 0.33; Δχ²(*df* = 1) = 14.22; *P* < 0.001 for older adults, in line with its role in memory processing^100^. Notably, when we specified a second, multiple regression model that searches for unique effects, we found that the LC was still reliably associated with episodic memory performance, *β* = 0.43; Δχ²(*df* = 1) = 11.96; *P* < 0.001 for older adults, whereas MTL volumes were not, *β* = 0.16; Δχ²(*df* = 1) = 2.46; *P* = 0.117 for older adults (Fig. 6 and Supplementary Fig. 13), when accounting for the respective other regions (for example, controlling for SN-VTA and MTL volume when evaluating the association between LC and episodic memory). Similarly, the SN-VTA was related to working memory after controlling for MTL volumes and LC integrity, *β* = 0.28; Δχ²(*df* = 1) = 5.8; *P* = 0.016 for older adults. For similar analyses that are based on intensity values averaged across the LC or SN-VTA, see Supplementary Table 7. Taken together, our results suggest a robust association of catecholaminergic nuclei and memory that cannot be fully accounted for by MTL volume.

### Longitudinal changes in LC integrity predict future episodic memory performance

Cross-sectional studies point to late-life differences in catecholaminergic nuclei^59,66–68,79,101^; however, longitudinal data showing within-person changes are scarce. Thus, we combined imaging data of the two timepoints (TP1–TP2; mean delay ~1.9 years) to test for individual changes in LC and SN-VTA integrity estimates in later life^78,102^.

**Fig. 7:**
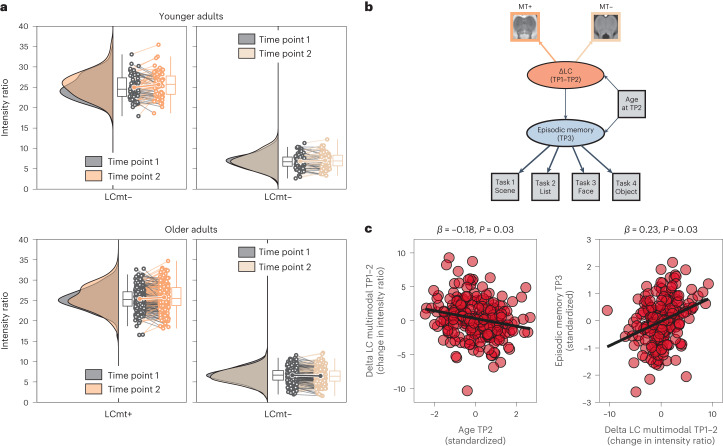
Longitudinal changes in LC intensity ratios and their association with age and future memory performance in older adults. **a**, Numerically, older adults show more negative average change in LC intensity across timepoints compared to younger adults. MRI sequences include an MT sequence, acquired once with a dedicated magnetic saturation pulse (MT+) and once without, resulting in a proton density image (MT−). For the FSE sequence, only cross-sectional data are available. **b**, Schematic depiction of the structural equation model probing the association of longitudinal change in multimodal LC integrity with future episodic memory performance, accounting for chronological age. For the full model, see Supplementary Fig. 29. **c**, Scatter plots showing (1) more negative LC change in older adults of higher age and (2) a prediction of future memory performance based on LC change (controlling for chronological age). For similar analyses using SN-VTA and working memory data, see Supplementary Figs. 30 and 31. Raincloud plots are based on ref. ^146^. *n* = 320 biologically independent participants. Statistics are based on two-sided likelihood-ratio tests without additional adjustment for multiple comparisons. For full test statistics, see Supplementary Table 3. Box plots are defined by the following values: lower and upper bounds of the box, quartiles (0.25 (Q1) and 0.75 (Q3)); center of the box, quartile 0.5 (Q2); lower whisker (Q1 − 1.5 × interquartile range); upper whisker (Q3 + 1.5 × interquartile range).

First, we observed that change in the catecholaminergic nuclei was correlated across imaging modalities (MT+ and MT−; no longitudinal FSE data available), suggesting that the different MR sequences pick up a common underlying construct (that is, change in catecholaminergic nuclei), *r* = 0.16; Δχ²(*df* = 1) = 6.09; *P* = 0.014 for older adult LC; *r* = 0.13; Δχ²(*df* = 1) = 5.91; *P* = 0.015 for older adult SN-VTA (Supplementary Figs. 16 and 21). Thus, we again integrated across sequences to retrieve multimodal latent change factors for LC and SN-VTA integrity (Supplementary Figs. 17 and 22). For both catecholaminergic systems, we found reliable individual differences in change, Δχ²(*df* = 1) = 6.09; *P* = 0.014 for older adult LC; Δχ²(*df* = 1) = 5.91; *P* = 0.015 for older adult SN-VTA. However, there was no reliable mean change at the group level in either nucleus, *P* > 0.1 in older adults. That is, we observed that older adults differed from one another in how their LC and SN-VTA changed over time; although some older adults showed increases in intensity ratios, others showed decreases (Fig. 7)^59,66^. Control analyses indicated that changes in neuromodulatory integrity were not associated with the spatial positions from which intensity ratios were sampled at TP1 and TP2, making movement in the scanner an unlikely explanation for individual differences in change (Supplementary Fig. 32).

Finally, to test the behavioral implications of these late-life changes in catecholaminergic nuclei, we probed whether our neural change model (TP1–TP2) could be used to predict future cognition (at TP3, using the previously established cognitive factor structure (compare to Supplementary Fig. 9)). We additionally included chronological age (at TP2) as a predictor, to test and account for potential age effects in rate of decline (Supplementary Figs. 29 and 30).

Note that, here, we related changes in catecholaminergic nuclei to subsequent cognitive performance (at TP 3), as our analyses indicated task-specific memory changes (Methods and Supplementary Results).

In line with a late-life degeneration of neuromodulatory centers^6,8,9,59^, we found more negative change in the LC and SN-VTA with increasing age, *β* = –0.18; Δχ²(*df* = 1) = 4.81; *P* = 0.028 for older adult LC; *β* = –0.29; Δχ²(*df* = 1) = 3.95; *P* = 0.047 for older adult SN-VTA. Moreover, we observed that changes in LC integrity predicted subsequent episodic memory performance over and above chronological age, *β* = 0.23; Δχ²(*df* = 1) = 4.73; *P* = 0.03 for older adult LC (Fig. 7); *β* = 0.27; Δχ²(*df* = 1) = 1.55; *P* = 0.213 for older adult SN-VTA (Supplementary Fig. 31). Taken together, our results are in line with the proposition that a decline of the LC in later life is associated with diminished episodic memory performance^8,59,79,103^.

## Discussion

This study sought to disentangle the effects of declining dopaminergic and noradrenergic neuromodulation on late-life memory. We took advantage of a multimodal imaging protocol and extensive cognitive assessments across several timepoints to contrast the behavioral implications of LC and SN-VTA integrity.

We found that different imaging approaches for catecholaminergic nuclei (FSE, MT+ and MT−) show a high agreement. Thus, we used latent-variable modeling to integrate across MRI modalities and retrieve multimodal LC and SN-VTA integrity factors that were significantly more reliable than their individual components. After establishing reliable in vivo integrity proxies, we probed their associations with late-life cognition. We used an extensive neuropsychological test battery and a previously identified factor structure to demonstrate that these two catecholaminergic systems, although positively coupled, differ in their relationship to three domains of aging cognition: episodic memory, working memory and fluid intelligence.

We observed a general (that is, task and imaging sequence independent) yet specific association of LC integrity and late-life episodic memory performance (that is, stronger for episodic memory as compared to working memory and fluid intelligence). By contrast, SN-VTA integrity was linked to better working memory. Remarkably, both associations remained reliable even after accounting for the respective other neuromodulatory system and a key node in the memory network, the MTL, suggesting robust effects. Corroborating this interpretation, associations between catecholaminergic integrity and late-life memory performance were qualitatively unchanged when including participants’ age, education and sex as covariates (Supplementary Results).

Leveraging the longitudinal nature of this dataset, we also investigated late-life changes in the LC and SN-VTA over a delay of approximately 2 years. A principal finding was that older age was associated with more negative change in each catecholaminergic system, in line with the late-life degeneration of neuromodulatory centers. Moreover, we showed that changes in the LC predicted future episodic memory performance over and above chronological age and education (Supplementary Results). Taken together, this study suggests that dopaminergic and noradrenergic neuromodulation play domain-specific roles in determining the trajectory of cognition in later life and provides insights into the neural basis of human senescent memory decline.

The loss of dopaminergic neuromodulation has long been recognized as a crucial determinant of late-life cognitive deficits^6,7^. More recently, technical advances have also facilitated studies of the noradrenergic LC^48^ that long seemed unattainable^44,45^. We used two types of imaging sequences (FSE and MT+) validated on postmortem specimens of the LC^49^ and SN-VTA^50,51^. Neuromelanin, an insoluble catecholamine-derived pigment that traps metals and exhibits paramagnetic properties, is thought to contribute to the hyperintensity of catecholaminergic nuclei^14,48,50^. Moreover, the high density of water protons and paramagnetic ions in large catecholaminergic neurons has also been proposed as the source of their MRI contrast^55,56^. In line with this, we provide a quantification of the LC and SN-VTA based on a proton density-weighted sequence (that is, without dedicated MT preparation pulse (MT−)). MT imaging studies frequently estimate a ratio score based on sequences with and without dedicated preparation pulse (that is, MT+ and MT−)^104^. However, the sensitivity of our MT− sequence for the LC and SN-VTA suggests that this ratio would reduce the detectability of these nuclei. Notably, we observed that, despite differences in mean contrast, LC and SN-VTA hyperintensities were correlated across all imaging modalities (FSE, MT+ and MT−), suggesting that they provide convergent information about the same underlying construct (that is, catecholaminergic nuclei). We, thus, leveraged our multimodal approach to estimate latent factors for catecholaminergic nuclei integrity based on the commonalities across imaging sequences while removing modality-specific measurement error^75,76^. Moreover, using data from both imaging timepoints, we show that semiautomatic analyses of the intensity of catecholaminergic nuclei have high reliability, especially when multimodal assessments are available^105^. The retention of salient experiences is enhanced by catecholamine release in the hippocampus, which facilitates synaptic plasticity and memory^32,34,40^. Although the SN-VTA has long been attributed as the source of the memory-enhancing dopaminergic inputs^32,35,39^, recent findings point to a denser innervation by the LC that can provide noradrenergic as well as dopaminergic signals^38,40^. Here, we compared the association of the two catecholaminergic centers with an extensive set of tasks that are thought to depend more (episodic memory) or less (working memory) on hippocampal processing^100,106^. We observed that LC integrity was specifically related to late-life episodic memory performance (as compared to working memory or fluid intelligence) and that this association was stronger than the SN-VTA–episodic memory relationship. Although we observed similar associations between neuromodulatory integrity and memory performance when comparing age groups, the lower sample size for younger adults^57^ may have limited our ability to detect effects in this group alone. Mechanistically, our finding of an LC–episodic memory association might be explained by a catecholaminergic modulation of hippocampal synaptic plasticity^37,40,91^, but our data cannot rule out other memory-related mechanisms, such as gain modulation^7,21–24^ and prefrontal delay activity^10,27^. Our observations are supported by a series of large-scale in vivo imaging studies that showed reliable LC–cognition associations in aging^59,79,89,90^ and particularly with episodic memory^59,79,107^. In addition, they concord with a recent report linking anteromedial and superior substantia nigra intensity to attentional performance^108^, a cognitive concept overlapping with working memory^22^.

Animal research suggests also a noradrenergic role in attentional processes^10,22,109^, particularly in tasks that require attentional re-orienting^110,111^. By contrast, the working memory indicator tasks used in the current study (for example, number n-back task) require participants to hold information active in mind and may, thus, depend less on noradrenergic neuromodulation (but see ref. ^29^). Interestingly, time-resolved measures associated with phasic LC activity (such as pupil dilation and the P300 event-related potential^23,112–114^) show consistent associations with individual differences in attentional performance^115,116^, calling for more multimodal research.

Cross-sectional studies point to late-life differences in catecholaminergic nuclei^66–68,79,101^. Here, we provide a characterization of late-life longitudinal changes in the LC and SN-VTA. In general agreement with extrapolations from cross-sectional data, we found more negative change in both catecholaminergic systems with increasing age. Subcortical neuromodulatory centers, such as the LC and the SN-VTA, are among the first sites to accumulate pathology in age-associated diseases, such as Alzheimer’s^11^ and Parkinson’s^63^, and show severe degeneration with disease progression^13,117^. In combination with earlier work suggesting that lower catecholaminergic contrast with advancing age might be linked to impending pathology^58,61,69,70,108^, our findings may indicate subthreshold pathological processes in a subset of our older participants. This interpretation is supported by our prediction of poorer future memory performance in individuals with more negative LC change^103^. That is, we tested the association between individual changes in LC integrity with the (cross-sectional) level of future episodic memory performance, as our analyses indicated task-specific memory changes (Methods and Supplementary Results). In line with the observed relation between LC changes and future memory performance, a recent meta-analysis demonstrates the efficacy of noradrenergic treatments in improving cognitive symptoms in Alzheimer’s disease^118^. Mirroring its clinical potential, MRI-indexed catecholaminergic integrity has been suggested as a useful tool for stratifying patients suffering from neurodegenerative diseases in clinical trials that include noradrenergic treatments^48,119^. Some older participants also showed LC and SN-VTA intensity increases over time, which might indicate higher intracellular proton density^56^, potentially linked to the activity-related volume increase of catecholaminergic cells^9,12,120,121^. We cannot rule out that also non-biological factors, such as measurement noise, may have contributed to intensity increases over time. However, our multimodal analysis approach should attenuate its influence compared to unimodal analyses^78^. Future quantitative MRI assessments^122^ of neuromodulatory integrity may overcome the need to standardize the intensity of catecholaminergic nuclei to a reference region^48^ (Methods) and, thus, exclude potential confounding effects.

The present study highlights the utility of multimodal longitudinal assessments of catecholaminergic nuclei to elucidate the neurobiological basis of senescent memory decline. We dissociated the roles of the noradrenergic LC and dopaminergic SN-VTA in late-life cognition. While the former showed robust associations with current and future episodic memory performance, the latter showed a relationship with working memory performance. These differential relationships between dopaminergic and noradrenergic nuclei and late-life cognition have potential implications for age-associated diseases that affect these nuclei, such as Alzheimer’s and Parkinson’s^11,59,63^. Furthermore, accurate longitudinal assessments of catecholaminergic nuclei may provide early markers predicting cognitive decline.

## Methods

### Study design and participants

Data were collected as part of the Berlin Aging Study II (BASE-II), an ongoing longitudinal study that investigates neural, cognitive, physical and social conditions related to successful aging (for more information, see https://www.base2.mpg.de/en, refs. ^71–74^ and Supplementary Methods). Cognitive performance was assessed in three time periods (TP1–TP3) between 2013 and 2020 (TP1: 2013–2015; TP2: 2015–2016; TP3: 2018–2020) with a mean duration between cognitive assessments of 2.246 years (TP1–TP2; s.d.: 0.603) and 2.917 years (TP2–TP3; s.d.: 0.438).

A subset of BASE-II participants also underwent MRI. Eligible participants had no history of neurological or psychiatric disorders or head injuries and did not take medication that may affect memory function. Imaging data were collected in two time periods (TP1 and TP2) in temporal proximity to the cognitive assessments (mean delay between MRI waves, 1.894 years; s.d.: 0.656). Participants were considered for further analyses only if at least one type of imaging sequence sensitive for dopaminergic or noradrenergic neuromodulatory centers was available (see below). For TP1, this corresponds to 288 participants out of a total of 488 participants with imaging data, whereas, for TP2, this corresponds to 320 out of 323 participants with imaging data. Thus, our analyses included a total of 320 individual participants. Although not all imaging sequences were available for all participants (Supplementary Table 1), 203 participants have relevant MRI data for both TP1 and TP2.

The final sample (*n* = 320) included 69 younger adults (22 female; mean age (s.d.): 32.705 (3.884) years (at TP2)) and 251 older adults (91 female; mean age (s.d.): 72.414 (4.045) years (at TP2)). Sample descriptives are reported in Table 1.

**Table 1:** Summary of sample descriptives for younger and older adults

	Younger adults (*n* = 69; 22 female)		Older adults (*n* = 251; 91 female)	
	Mean (s.d.)	Range	Mean (s.d.)	Range
Age (years, at TP2)	32.705 (3.884)	25.414–40.203	72.414 (4.045)	62.534–83.162
Education (years) (available for *n* = 285)	14.242 (2.519)	10–18	14.08 (2.924)	7–18
Mini Mental State Examination (available for *n* = 204)	–	–	28.613 (1.336)	24–30

Note: Mini Mental State data were assessed around TP3, during a medical examination at a different visit as part of the longitudinal BASE-II.

The cognitive and imaging assessments were approved by the institutional review boards of the Max Planck Institute for Human Development and the German Psychological Society, respectively. Participants provided written informed consent and were reimbursed for their participation.

### Cognitive data assessment

At TP1–TP3, cognition was tested using a comprehensive computerized battery probing key cognitive functions. Performance was assessed in small groups of 4–6 participants. Cognitive test sessions lasted approximately 3.7 h and included 16 tasks (at TP2); of these, three measured working memory, four measured episodic memory, and three measured fluid intelligence^77^. Although the exact composition of the cognitive assessments changed across waves, the same tasks were used to measure working memory, episodic memory and fluid intelligence at TP1–TP3. Only older adults were tested at TP3. For a detailed task description, see refs. ^77,123,124^; below, we provide a brief overview of the measures relevant to the current analyses.

### Working memory assessment

#### Spatial updating (abbreviated as ‘spatial-u’)

Participants were shown a display of 2–3 3 × 3 grids, in each of which a blue dot was presented in one of the nine tiles. Participants were asked to memorize the locations of the blue dots and mentally update them according to shifting operations that were indicated by arrows appearing below the dots. Six updating operations were required before the 3 × 3 grids reappeared and participants indicated the end position of the blue dots (by mentally combining their start position and the six shifting operations). We used the number of correct placements as an indicator of working memory performance^77,124^.

#### Letter updating (abbreviated as ‘letter-u’)

Participants were shown a sequence of 7–13 letters. Once the presentation ended, they were asked to report, in correct order, the last three letters that were shown. The number of correctly reported letters was used as a measure of working memory performance^77^.

#### Number n-back (abbreviated as ‘number’)

Three digits (1–9) were presented consecutively in three adjacent cells, followed by the next sequence of three digits. Participants indicated by button press whether the currently presented digit matched the digit shown three steps before^77,124^. We took participants’ accuracy as an indicator of their working memory performance.

### Episodic memory assessment

#### Scene encoding (abbreviated as ‘scene’)

Participants incidentally encoded 88 scene images by performing indoor/outdoor judgments on each image. The encoding phase was followed by an old/new recognition memory test, which included confidence judgments. Recognition memory was tested after a delay of approximately 2.5 h and served as episodic memory performance index (hits − false alarms)^76,77,79^.

#### Verbal learning and memory task (abbreviated as ‘list’)

Participants first learned a 15-word list that was presented via headphones. The task comprised five learning trials, each followed by a free-recall period in which participants entered the words that they remembered via keyboard (trials 1–5; recall of learning list). After these initial learning–recall cycles, participants were presented an interference list, and their delayed recall and recognition memory was assessed. The sum of correctly recalled words during the learning–recall cycles (trials 1–5) served as an episodic memory measure^76,77,79^.

#### Face–profession task (abbreviated as ‘face’)

Participants studied 45 pairs of face images and profession words. The tasks consisted of an incidental encoding phase, a 2-min distraction phase and, finally, a recognition memory task including old, new and rearranged face–profession pairs. Corrected recognition memory scores for rearranged pairs were used as the performance index.

#### Object–location task (abbreviated as ‘object’)

Participants encoded the location of 12 digital photographs of real-life objects on a 6 × 6 grid. After encoding, the objects reappeared next to the grid, and participants were instructed to reproduce their correct location by placing the items in the grid. The sum of correct placements served as the index of episodic memory.

### Fluid intelligence assessment

#### Practical problems (abbreviated as ‘problem’)

Participants were sequentially presented 18 items depicting everyday problems (for example, the hours of a bus timetable and a subway map), in order of ascending difficulty. For each of these problems, five response alternatives were provided, and participants selected the correct option by clicking on it. We took the sum of correctly solved problems as the measure of fluid intelligence^77,123^.

#### Figural analogies (abbreviated as ‘analog)

Participants were instructed to draw analogies. They were presented with 22 items in ascending difficulty that followed the format ‘*A* is to *B* as *C* is to *?*’. Below each item, five response alternatives were presented, and participants selected the correct option by clicking on it. The sum of correctly given answers served as the index of fluid intelligence^77,123^.

#### Letter series (abbreviated as ‘letter’)

Participants were shown 22 series of five letters, each ending with a question mark (for example, c-e-g-i-k-?). Each series followed a simple rule (for example, +1, −1, +2 or +2, −1), with increasing difficulty. Below each letter series, five response alternatives were presented, and participants selected the correct option by clicking on it. The sum of correct responses served as the fluid intelligence measure^77,123^.

All tasks included practice blocks to familiarize participants with the instructions. Note that these tasks have previously been used to estimate latent factors of working memory, episodic memory and fluid intelligence^77^ (also see refs. ^76,79,125^).

### MRI data assessment

To investigate the associations of dopaminergic and noradrenergic integrity with late-life cognition, younger and older participants underwent 3T MRI at TP1 and TP2 (MAGNETOM TIM Trio, Siemens Healthcare). Only those sequences used in the current analyses are described below. The imaging protocol included three scans sensitive for the SN-VTA and LC: an FSE sequence (sometimes also called Turbo Spin Echo) and an MT sequence, acquired once with a dedicated magnetic saturation pulse (MT+) and once without, resulting in a proton density image (MT−). Moreover, a Magnetization-Prepared Gradient Echo (MPRAGE) sequence was collected to facilitate co-registration to standard space and to estimate volumes for regions of interest. Moreover, the MPRAGE sequence was used during acquisition to align the FSE sequence perpendicularly to the plane of a participant’s brainstem. Note that, for some participants, specific absorption rate limits were exceeded during the FSE acquisition, as reported previously^79^ (also see refs. ^47,126^). Sequence parameters are reported in Supplementary Table 2.

### MRI data analysis

We applied a previously established semiautomatic analysis procedure to extract individual LC and SN-VTA intensity values from structural imaging data (for a detailed description and validation, see ref. ^79^; for applications, see refs. ^58,80,81^; for an independent validation, see ref. ^82^). The following procedure was performed separately for TP1 and TP2 imaging data.

### Template construction and standardization

First (step 1), scans of each scan modality (MPRAGE, FSE, MT+ and MT−) were iteratively aligned across participants using a template-based procedure implemented in Advanced Normalization Tools (ANTs) (version 2.3.3^127,128^; antsMultivariateTemplateConstruction, six iterations, including N4BiasFieldCorrection). A schematic visualization of the procedure is included in Supplementary Methods. Before template construction, MPRAGE and MT− scans were resampled to 0.5-mm isometric resolution (ANTs’ ResampleImage). Moreover, to facilitate template construction, participants’ native-space FSE scans were aligned to their template-space MPRAGE scans (antsRegistrationSyNQuick)^79^. Native-space MT+ scans were aligned to resampled MT− scans to account for potential movement effects between scan acquisitions (antsRegistrationSyNQuick). After their alignment, MT− and MT+ scans were submitted to a common multimodal template construction, whereas FSE and MPRAGE scans each were used to generate a brainstem and whole-brain template, respectively.

Next (step 2), modality-specific group templates (MPRAGE, FSE, MT+ and MT−) were linearly and nonlinearly co-registered (antsRegistration) to standard space (MNI-ICBM 152 linear, 0.5 mm^129^). Specifically, templates with a sensitivity for catecholaminergic nuclei (FSE, MT+ and MT−) were first standardized to the whole-brain MPRAGE template (using a co-registration mask). Next, the MPRAGE template was co-registered to MNI space, and the transformations were applied to the other templates (FSE, MT+ and MT−; antsApplyTransforms). To improve co-registration accuracy, whole-brain templates (MPRGAE, MT− and MNI) were skull stripped before alignment using the FMRIB Software Library (bet2, FSL version 5)^130^.

Finally (step 3), all transformation matrices were concatenated and applied to individual participants’ scans to bring them from native to MNI space in a single step (antsApplyTransforms).

### Semiautomatic intensity assessment

To extract the intensity values of catecholaminergic nuclei, in standard space, individual scans were masked with binary volumes of interest using Statistical Parametric Mapping toolbox version 12 (SPM12, version 6685)^131^ in MATLAB (MathWorks). In particular, for the LC, we applied a previously established high-confidence consensus mask^58^. For the SN-VTA, we relied on a previously established mask that was based on manual tracings in template space^50^. Inter-participant comparisons of arbitrality scaled MRI intensity values require that intensity values are normalized within participants^48^. Thus, we also masked scans (FSE, MT+ and MT−) with volumes of interest in potine^58^ and midbrain^50^ reference areas, in line with earlier research^47,50,79^. Note that the fourth ventricle, which is in close proximity to the LC, appears hyperintense in our MT+ scans. Thus, to rule out that the hyperintensity of the ventricle could interfere with automatized LC assessments, we generated a sample-specific ventricle volume-of-no-interest (based on the MT– group template), which we removed from MT+ and MT− scans before value extraction (templates and ventricle mask are available from ref. ^84^). Within the masked scans (FSE, MT+ and MT−), we then automatically searched for the voxel of highest intensity in the LC, SN-VTA and reference regions. Next, for each participant, spatially resolved intensity ratios for the LC and SN-VTA were computed per hemisphere (left, right) on a slice-by-slice basis using the following formula^47,79^:

$${LC}_{Ratio}=\frac{{{\max }}\left({ROI}\,\right)-{{\max }}\left({Ref}\,\right)}{{{\max }}({Ref}\,)}$$

where max(*ROI*) denotes the peak intensity for a given slice in the LC or SN-VTA regions of interest and max(*Ref*) indicates the peak intensity in the respective reference region. For the FSE modality, two scans were available per participant (see ‘MRI data assessment’ subsection), and we averaged the extracted intensity ratios within participants to obtain more stable estimates. For further analyses, for all modalities (FSE, MT+ and MT−), the peak intensity ratio across the regions of interest (LC and SN-VTA) was calculated as an overall integrity measure^45,58,79^. Outlier values exceeding ±3 s.d. were dropped, whereas all other values were linearly scaled (×100) to facilitate subsequent model estimation. Note that all analyses, including LC or SN-VTA data, were based on peak intensity ratios. That is, the peak intensity of catecholaminergic nuclei standardized using nearby white matter regions (not their raw intensity values). To facilitate readability, we will nonetheless use the term ‘intensity’ in our description.

At acquisition, the FSE sequence was centered on the pons and contained fewer slices compared to the MT+ and MT− sequences. As evident in Fig. 2a, the most rostral slices of our volume of interest (LC meta mask and reference mask) reach the edges of the brainstem template and include high-intensity artifacts, which, however, are reliably excluded from analyses using the semiautomatic procedure (see description of peak detection above).

### Statistics and reproducibility

We used structural equation modeling to relate multimodal brain and cognitive data. In particular, the significance of all parameters of interest was evaluated using likelihood-ratio tests (for details, see below). No statistical methods were used to pre-determine sample sizes, but our sample sizes are similar to those reported in previous publications^59,79^. Based on visualizations of data distributions (compare, for example, to Figs. 3 and 5), we assumed normally distributed cognitive and neural data, but this was not formally tested. Randomization and blinding were not possible in this longitudinal aging study.

During the semiautomatic intensity assessment, outlier values exceeding ±3 s.d. were dropped from further analyses. Outliers were also excluded for voxel-based morphometry data (see below) using the same cutoff.

### Structural equation modeling

We used structural equation modeling to evaluate inter-individual and intra-individual differences in catecholaminergic nuclei and their association with cognition using the Ωnyx software environment (versions 1.0-1026–1.0-1040)^132^ and the lavaan R package (versions 0.6-6–0.6-14)^133^. All models used full information maximum likelihood estimation to account for missing values. The adequacy of the reported models was evaluated using *χ*^2^ tests (that is, an absolute fit index) as well as two frequently reported approximate fit indices: the root mean square error of approximation and the comparative fit index. Root mean square error of approximation values close to or below 0.06 and comparative fit index values close to 0.95 or greater indicate good model fit^134,135^. Unless otherwise noted, multi-group models were fit, comprising sub-models for younger and older adults. For this, invariance across age groups was evaluated by a hierarchical series of likelihood-ratio tests, probing group differences in (1) factor loadings (weak invariance), (2) indicator intercepts (strong invariance) and (3) residual variances (strict invariance)^136^. In the case of longitudinal models, the same criteria were applied to test invariance across time^102^. After establishing adequate model fit and invariance, the significance of parameters of interest was evaluated using likelihood-ratio tests. That is, we created two nested models—in one model, the parameter of interest was freely estimated, whereas, in the other model, it was fixed to zero. If fixing the parameter of interest to zero resulted in a drop in model fit, as evaluated using a likelihood-ratio test comparing the two nested models, this indicated the significance of the parameter^137^. We used an alpha level of 0.05 for all statistical tests. Statistical results with *P* values between 0.05 and 0.1 are described as a statistical trend. All analyses are based on two-sided statistical tests and did not include corrections beyond those mentioned in the respective sections. In the following, cross-sectional models refer to models that include TP2 data only, whereas longitudinal models evaluate the change in parameters of interest over time (TP1–TP2 or TP1–TP3). Model code, visualizations and output are available from ref. ^84^.

### Cross-sectional cognitive models

We made use of a previously established factor structure^77^ to integrate performance across several working memory, episodic memory and fluid intelligence tasks (see ‘Cognitive data assessment’ subsection) and capture their shared variance on a latent level. Latent variables account for measurement error in the observed scores (cognitive tasks) and, thus, increase statistical power to detect true effects^78^.

We added covariances among the latent working memory, episodic memory and fluid intelligence factors, as performance across these cognitive domains had been shown to be correlated^77^ (Supplementary Fig. 9).

### Cross-sectional neural models

We also adapted a previously established factor structure^79^ to capture LC and SN-VTA intensity on a latent level. Specifically, for each region (LC and SN-VTA) and scan modality (FSE, MT+ and MT−), we used the left and right hemispheric peak intensity as observed scores to estimate a modality-specific integrity factor on a latent level. Note that our FSE sequence covered only the brainstem. Thus, we cannot obtain SN-VTA estimates for this scan modality. To test the agreement in integrity estimates across modalities (FSE, MT+ and MT−), we added covariances among the modality-specific latent factors for each brain region (Supplementary Fig. 3).

Using the model described above, we found a high correspondence in the integrity estimates for each nucleus across modalities (Results). Thus, in a second model, we introduced a multimodal integrity factor for the LC and SN-VTA that captures the commonalities across scan modalities while removing the modality-specific measurement error (for similar approaches and a detailed discussion, see refs. ^75,76^ and Supplementary Fig. 4). Finally, as dopamine and noradrenaline are products of the same biosynthesis pathway, with dopamine a direct precursor to noradrenaline^88^, we evaluated the association of the multimodal LC and SN-VTA integrity factors by estimating their covariance.

### Cross-sectional neurocognitive models

After separately establishing models for our cognitive and neural measures that showed acceptable fit and invariance across age groups, we unified these models to probe associations between catecholaminergic nuclei and cognition^79^. In the unified neurocognitive model, we estimated covariances between the multimodal LC and SN-VTA integrity factors and working memory, episodic memory and fluid intelligence (correlation model; Supplementary Figs. 10 and 12). In addition, we specified a second neurocognitive model, in which regression paths were drawn from the latent neural to the cognitive factors (regression model; Supplementary Figs. 11 and 13). The correlation model evaluates associations between the LC and cognition irrespective of those between the SN-VTA and cognition. By contrast, the regression model tests whether one region explains variance in the cognitive factors over and above the other region, thus providing complementary information^76^.

### Longitudinal cognitive models

Making use of the repeated assessments of cognitive performance, we tested for late-life changes in working memory and episodic memory tasks over time (TP1–TP3). Note that cognitive data at TP3 were available only for older adults (see ‘Cognitive data assessment’ subsection). Thus, here, we relied on single-group models that excluded younger adults. In particular, we specified a latent change score model^78,138^ for each cognitive task. These latent change score models yield a latent slope factor for each task that expresses participants’ performance difference between TP1 and TP3 (Supplementary Figs. 25 and 26).

Next, we tested whether there is a common latent factor of working memory or episodic memory change. If changes in performance were shared across tasks of one cognitive domain (that is, task independent), they could be captured on a higher-order latent level (compare to the latent working memory and episodic memory factors in the cross-sectional cognitive model; Supplementary Fig. 9). To evaluate potential associations in the changes across working memory and episodic memory tasks, we added covariances among the task-specific slope terms (Supplementary Figs. 25 and 26).

Most covariances across task-specific slope terms did not reach significance (Supplementary Results). Thus, we did not further attempt to capture task-independent changes in working memory or episodic memory on a latent level.

### Longitudinal neural models

#### Assessment of across-time stability

Our cross-sectional analyses of different MRI sequences sensitive for catecholaminergic nuclei (FSE, MT+ and MT−) demonstrated a high agreement in integrity estimates across imaging modalities (see ‘Cross-sectional neural models’ subsection, Results and Fig. 3). Leveraging the longitudinal nature of this study, we additionally explored the stability of the modality-specific integrity estimates over time (TP1 and TP2), as a proxy for their reliability^85^. Specifically, we started with modality-specific SN-VTA and LC models for TP2 (see ‘Cross-sectional neural models’ subsection and Supplementary Fig. 3) and then appended the same variables for TP1. We introduced covariances linking modality-specific factors of TP1 and TP2 to evaluate the stability in integrity estimates over time (Supplementary Figs. 14 and 18). Moreover, we allowed for correlated residuals over time, as suggested for longitudinal models^102^.

Using the model described above, we found a high stability of the modality-specific integrity estimates for each region across time (Results, Fig. 3 and Supplementary Fig. 20). Similarly to our cross-sectional analyses, we, thus, again added multimodal integrity factors for the LC and SN-VTA for each timepoint (TP1 and TP2). If the multimodal integrity factors indeed remove measurement error^76^, we should observe a higher stability across timepoints of the multimodal as compared to the modality-specific integrity factors. To test this hypothesis, we computed the covariance between the multimodal factors of TP1 and TP2 (Supplementary Figs. 15 and 19). In addition, we directly compared the stability estimates, our reliability proxy, for the modality-specific and multimodal SN-VTA and LC factors. Note that the modality-specific and multimodal integrity models, including TP1 and TP2, were fit across younger and older adults to obtain a single reliability proxy for the complete sample.

### Assessment of within-person changes

Previous cross-sectional research observed between-person age differences in catecholaminergic nuclei integrity^66,67^; however, longitudinal studies that evaluate within-person changes in the SN-VTA and LC are scarce. Making use of the imaging data of both timepoints (TP1 and TP2), we, thus, estimated changes in catecholaminergic nuclei using latent change score models^78,138^ for each imaging modality (MT+ and MT−). Note that the FSE sequence was only acquired at TP2, precluding change analyses (Supplementary Table 1). To reduce model complexity, we averaged intensity ratios across hemispheres for these models. Similarly to our longitudinal cognitive analyses, we first evaluated whether the change in each region was shared across imaging modalities (that is, sequence independent) by computing the covariance of the modality-specific slope terms (Supplementary Figs. 16 and 21).

For the SN-VTA as well as the LC, we observed that the changes were indeed correlated across imaging modalities (MT+ and MT−). Thus, in a second set of models, we introduced a higher-order multimodal slope factor to capture the shared variance across modality-specific slope factors (Supplementary Figs. 17 and 22).

### Longitudinal neurocognitive models

In older adults, we found that SN-VTA and LC integrity changed across time (TP1–TP2; see ‘Longitudinal neural models’ subsection). To evaluate the behavioral implications of these changes, we leveraged a previously established cognitive factor structure^77,125^ (see ‘Cross-sectional cognitive models’ subsection). Specifically, we were interested in testing whether changes in catecholaminergic nuclei (TP1–TP2) could be used to predict future cognition (at TP3). For this, we unified our neural change models (Supplementary Figs. 17 and 22) with models of working memory and episodic memory (at TP3; compare to Supplementary Fig. 9). In the unified model, we specified regression paths from the neural change factors to the cognitive factors (SN-VTA to working memory; LC to episodic memory). Finally, we added chronological age (at TP2) as an additional predictor to test (1) whether catecholaminergic nuclei explains future cognition over and above age and (2) how changes in catecholaminergic nuclei differ in old age (Supplementary Figs. 29 and 30). Note that cognition at TP3 was assessed only for older adults. Thus, we specified single-group models excluding younger adults.

### Control analyses

Catecholaminergic neuromodulation influences neural processing in the MTL, a key node of the memory network^32,34,37,39,40,91^. Moreover, catecholaminergic nuclei have direct projections to the MTL^37,39,139,140^, and their integrity has been linked to tau pathology in these areas^58–60^. Thus, as a control analysis, we additionally evaluated MTL volumes (at TP2). This allowed us to compare our measures of catecholaminergic nuclei and their association with memory performance to those of a well-established player in the memory network^100^.

### Voxel-based morphometry assessment

Whole-brain MPRAGE images were processed using the voxel-based morphometry pipeline in SPM12 in MATLAB^131,141^. First, images were segmented into distinct tissue classes (for example, gray matter, white matter and cerebrospinal fluid) using SPM12’s unified segmentation procedure^142^. Next, a study-specific DARTEL template was created, and segmented images were aligned to the template, followed by spatial normalization, modulation and smoothing with a 2-mm full-width at half-maximum isotropic Gaussian kernel^143,144^. The resulting normalized, modulated and smoothed gray matter images were used to derive region-of-interest volumes. Region-of-interest masks from the AAL3 atlas were applied to processed gray matter images using native SPM functions and the get_totals script (http://www0.cs.ucl.ac.uk/staff/g.ridgway/vbm/get_totals.m) to calculate volumes for the parahippocampal and hippocampal regions^145^. Outlier values exceeding ±3 s.d. were dropped, whereas all other values were linearly scaled (×10,000) to facilitate subsequent model estimation. Before analyses, volume data were adjusted by dividing regional estimates by total intracranial volume.

### Cross-sectional neurocognitive models including voxel-based morphometry factor

Mimicking our integrity factors of catecholaminergic nuclei (Supplementary Fig. 3), we aggregated across left and right hemispheric volumes to estimate latent parahippocampal and hippocampal factors for older adults. We specified a covariance between the two regions to evaluate their association (Supplementary Fig. 5). We observed that parahippocampal and hippocampal factors were highly correlated (Supplementary Results). Thus, we introduced a higher-order MTL factor to capture their shared variance, which we then included in our neurocognitive models (see ‘Cross-sectional neurocognitive models’ subsection). Specifically, for older adults, we estimated one correlation model and one regression model^76^. In each model, we compared the associations of catecholaminergic nuclei and cognition with the MTL–cognition association (Fig. 6 and Supplementary Figs. 12 and 13). In addition, we quantified the interrelations of SN-VTA and LC integrity and MTL volume by estimating their covariance.

### Reporting summary

Further information on research design is available in the Nature Portfolio Reporting summary linked to this article.

## Supplementary information

Supplementary Information Supplementary Methods and Results.

Reporting Summary

## Data Availability

The data that our results are based on are available from the BASE-II steering committee upon approved research proposal (https://www.base2.mpg.de/en). For inquiries, contact Dr. L. Müller, BASE-II project coordinator (lmueller@mpib-berlin.mpg.de). To facilitate comparability of study results, we share the group templates with sensitivity for catecholaminergic nuclei (FSE, MT+, MT−) in MNI 0.5-mm linear space (https://osf.io/eph9a/)^84^. The LC consensus volume of interest (LC meta mask and pontine reference mask) is available at https://osf.io/sf2ky/ (ref. ^58^). We provide two synthetic datasets of simulated cases (*n* = 250) that follow the population described in our models, with the parameter values displayed in the model visualizations (generated using ref. ^132^). In combination with the model code (listed below), these data allow reproduction of key results.

## Code availability

All statistical models that our inferences are based on and their outputs are available at https://osf.io/eph9a (ref. ^84^). Vector graphics visualizations for all models are also available via the Open Science Framework (OSF) repository. Additional custom code used for processing behavioral and neural data are available from the corresponding author upon reasonable request.
