# Supplementary material for: The integrity of dopaminergic and noradrenergic brain regions is associated with different aspects of late-life memory performance
Source: Nat Aging. 2023 Aug 31;3(9):1128–43. doi: 10.1038/s43587-023-00469-z (PMC10501910; doi:10.1038/s43587-023-00469-z)
Supplement: Supplementary file 2 — Reporting Summary [file 43587_2023_469_MOESM2_ESM.pdf]

nature portfolio

Corresponding author(s): Martin J. Dahl (dahl@mpib-berlin.mpg.de)

Last updated by author(s): 2023/06/14

Reporting Summary

Nature Portfolio wishes to improve the reproducibility of the work that we publish. This form provides structure for consistency and transparency in reporting. For further information on Nature Portfolio policies, see our Editorial Policies and the Editorial Policy Checklist.

Statistics

For all statistical analyses, confirm that the following items are present in the figure legend, table legend, main text, or Methods section.

n/a	Confirmed	
☐	☒	The exact sample size (*n*) for each experimental group/condition, given as a discrete number and unit of measurement
☐	☒	A statement on whether measurements were taken from distinct samples or whether the same sample was measured repeatedly
☐	☒	The statistical test(s) used AND whether they are one- or two-sided<br>*Only common tests should be described solely by name; describe more complex techniques in the Methods section.*
☐	☒	A description of all covariates tested
☐	☒	A description of any assumptions or corrections, such as tests of normality and adjustment for multiple comparisons
☐	☒	A full description of the statistical parameters including central tendency (e.g. means) or other basic estimates (e.g. regression coefficient) AND variation (e.g. standard deviation) or associated estimates of uncertainty (e.g. confidence intervals)
☐	☒	For null hypothesis testing, the test statistic (e.g. *F*, *t*, *r*) with confidence intervals, effect sizes, degrees of freedom and *P* value noted<br>*Give P values as exact values whenever suitable.*
☐	☒	For Bayesian analysis, information on the choice of priors and Markov chain Monte Carlo settings
☐	☒	For hierarchical and complex designs, identification of the appropriate level for tests and full reporting of outcomes
☐	☒	Estimates of effect sizes (e.g. Cohen's *d*, Pearson's *r*), indicating how they were calculated

Our web collection on statistics for biologists contains articles on many of the points above.

Software and code

Policy information about availability of computer code

Data collection	-
Data analysis	Imaging analyses used SPM12 (v6685), ANTs (v2.3.3), and FSL (v5).<br>We used structural equation modeling (SEM) to evaluate inter- and intra-individual differences in catecholaminergic nuclei and their association with cognition using the Ωnyx software environment (v 1.0-1026–1.0-1040) and the lavaan R package (v0.6-6–0.6-14). All statistical models that our inferences are based on and their outputs are available via: https://osf.io/eph9a (DOI 10.17605/OSF.IO/EPH9A)

For manuscripts utilizing custom algorithms or software that are central to the research but not yet described in published literature, software must be made available to editors and reviewers. We strongly encourage code deposition in a community repository (e.g. GitHub). See the Nature Portfolio guidelines for submitting code & software for further information.

Data

Policy information about availability of data

All manuscripts must include a data availability statement. This statement should provide the following information, where applicable:

- Accession codes, unique identifiers, or web links for publicly available datasets
- A description of any restrictions on data availability
- For clinical datasets or third party data, please ensure that the statement adheres to our policy

The data that our results are based on are available from the BASE-II steering committee upon approved research proposal (see https://www.base2.mpg.de/en). For

inquiries, please contact Dr. Ludmilla Müller, BASE-II project coordinator (lmueller@mpib-berlin.mpg.de).
To facilitate comparability of study results, we share the group templates with sensitivity for catecholaminergic nuclei (FSE, MT+, MT–) in MNI 0.5 mm linear space (https://osf.io/eph9a/).
The LC consensus volume of interest (LC meta mask and pontine reference mask) is available via https://osf.io/sf2ky/ .
We provide two synthetic datasets of simulated cases (n = 250) that follow the population described in our models, with the parameter values displayed in the model visualizations (generated using Onyx). In combination with the model code (see below), these data allow reproduction of key results.

Human research participants

Policy information about studies involving human research participants and Sex and Gender in Research.

Reporting on sex and gender	Our main analyses are not split by sex or gender as this was not our research question. For a recent publication that addressed these questions in a partly overlapping sample, see: https://doi.org/10.1016/j.neurobiolaging.2020.12.019 However, we added analyses split by sex to the supplementary information
Population characteristics	The final sample (n = 320) included 69 younger adults (22 female; mean age (SD): 32.705 (3.884) years [at TP2]) and 251 older adults (91 female; mean age (SD): 72.414 (4.045) years [at TP2]). Sample descriptives are reported in Table 2.
Recruitment	Data were collected as part of the Berlin Aging Study-II (BASE-II), an ongoing longitudinal study that investigates neural, cognitive, physical, and social conditions related to successful aging (for more information, see https://www.base2.mpg.de/en and references:<br><br>Delius, J.A.M. et al. (2015) Berlin Aging Studies (BASE and BASE-II). In Encyclopedia of geropsychology (Pachana, N. A., ed), pp. 386–395, Springer<br>Gerstorf, D. et al. (2016) The Berlin Aging Study II: An overview [Editorial]. Gerontology 62, 311–315<br>Bertram, L. et al. (2014) Cohort profile: The Berlin Aging Study II (BASE-II). Int. J. Epidemiol. 43, 703–712<br>Demuth, I. et al. (2021) Cohort profile: follow-up of a Berlin Aging Study II (BASE-II) subsample as part of the GendAge study. BMJ Open 11, e045576
Ethics oversight	The cognitive and imaging assessments were approved by the institutional review boards of the Max Planck Institute for Human Development and the German Psychological Society (DGPS), respectively. Participants provided written informed consent and were reimbursed for their participation.

Note that full information on the approval of the study protocol must also be provided in the manuscript.

Field-specific reporting

Please select the one below that is the best fit for your research. If you are not sure, read the appropriate sections before making your selection.

☒ Life sciences ☐ Behavioural & social sciences ☐ Ecological, evolutionary & environmental sciences

For a reference copy of the document with all sections, see nature.com/documents/nr-reporting-summary-flat.pdf

Life sciences study design

All studies must disclose on these points even when the disclosure is negative.

Sample size	No statistical methods were applied to determine sample size but our sample sizes are similar to those reported in previous publications (Dahl et al., 2019; Jacobs et al., 2021).<br>A subset of BASE-II participants underwent magnetic resonance imaging (MRI). Eligible participants had no history of neurological or psychiatric disorders, or head injuries, and did not take medication that may affect memory function. Imaging data were collected in two time periods (TP1, TP2) in temporal proximity to the cognitive assessments (mean delay between MRI waves 1.894 years; SD: 0.656). Participants were only considered for further analyses if at least one type of imaging sequence sensitive for dopaminergic or noradrenergic neuromodulatory centers was available (see Table1). For TP1, this corresponds to 288 participants out of a total of 488 participants with imaging data, whereas for TP2 this corresponds to 320 out of 323 participants with imaging data. Thus, our analyses included a total of 320 individual participants. While not all imaging sequences were available for all participants (see Table 1), 203 participants have relevant MRI data for both TP1 and TP2.
Data exclusions	All participants with relevant imaging and cognitive data were included in the analyses. That is, we did not exclude participants.<br>MRI-derived estimates that exceeded +/- 3 standard deviations (i.e., outliers) were replaced by NaN.
Replication	A direct empirical replication is not possible at this point because there is no comparable (longitudinal + multimodal) dataset. However, we attempted analytical replications. We demonstrate that brain–cognition associations hold across MRI-sequences and cognitive tasks (i.e., on a latent level), and across different statistical models (correlational model, multiple regression model including covariates).<br>We verify that the provided results are robust to the inclusion of several covariates (age, sex, education; see Supplementary Results) and across analytical procedures (Structural Equation Models [SEM]; Partial Least Squares Correlation [PLSC]; see Supplementary Results).<br>Findings were weaker when different metrics for neuromodulatory integrity were used (mean instead of peak intensity; see Supplementary Results).

Randomization	Randomization between experimental groups was not relevant in the current study as it did not include different experimental conditions. Participants were allocated to groups based on their age (i.e., non-random).
Blinding	Blinding was not relevant (and not possible) in the current study as it did not include different experimental conditions but different age groups. Identical computer code was used to perform the analyses in both age groups.<br>Staff involved in data collection was not involved in data analysis.

Reporting for specific materials, systems and methods

We require information from authors about some types of materials, experimental systems and methods used in many studies. Here, indicate whether each material, system or method listed is relevant to your study. If you are not sure if a list item applies to your research, read the appropriate section before selecting a response.

Materials & experimental systems

n/a	Involved in the study
☒	☐ Antibodies
☒	☐ Eukaryotic cell lines
☒	☐ Palaeontology and archaeology
☒	☐ Animals and other organisms
☒	☐ Clinical data
☒	☐ Dual use research of concern

Methods

n/a	Involved in the study
☒	☐ ChIP-seq
☒	☐ Flow cytometry
☐	☒ MRI-based neuroimaging

Magnetic resonance imaging

Experimental design

Design type	structural MRI
Design specifications	To investigate the associations of dopaminergic and noradrenergic integrity with late-life cognition, younger and older participants underwent 3T-MRI at TP1 and TP2 (MAGNETOM TIM Trio, Siemens Healthcare). Only those sequences used in the current analyses are described below. The imaging protocol included three scans sensitive for the SN–VTA and LC —a Fast Spin Echo sequence (FSE; sometimes also called Turbo Spin Echo [TSE]), and a Magnetization Transfer sequence, acquired once with a dedicated magnetic saturation pulse (MT+) and once without, resulting in a proton density image (MT–). Moreover, a Magnetization Prepared Gradient-Echo (MPRAGE) sequence, comparable to the ADNI protocol (www.adni-info.org), was collected to facilitate coregistration to standard space and to estimate volumes for regions of interest. Moreover, the MPRAGE sequence was used during acquisition to align the FSE sequence perpendicularly to the plane of a participant's brainstem. Note that for some participants specific absorption rate (SAR) limits were exceeded during the FSE acquisition, as reported previously. Sequence parameters are reported in Table 3.
Behavioral performance measures	no behavioral assessment within the scanner (i.e., structural sequences).

Acquisition

Imaging type(s)	structural
Field strength	3 T
Sequence & imaging parameters	See above for sequences. All MRI-sequence parameters are reported in Table 3.
Area of acquisition	brainstem (for FSE sequence), whole-brain for all other sequences

Diffusion MRI ☐ Used ☒ Not used

Preprocessing

Preprocessing software	Advanced Normalization Tools (ANTs, version 2.3)
Normalization	Magnetic resonance imaging data analysis:<br>We applied a previously established semi-automatic analysis procedure to extract individual LC and SN–VTA intensity values from structural imaging data (for a detailed description and validation, see: Dahl, M.J. et al. (2019) Rostral locus coeruleus integrity is associated with better memory performance in older adults. Nat. Hum. Behav. 3, 1203–1214). The following procedure was performed separately for TP1 and TP2 imaging data.<br><br>Template construction and standardization:<br>First (step 1), scans of each scan modality (MPRAGE, FSE, MT+ and MT–) were iteratively aligned across participants using a template-based procedure implemented in Advanced Normalization Tools [ANTs] (v. 2.3

antsMultivariateTemplateConstruction, 6 iterations, including N4BiasFieldCorrection). Before template construction, MPRAGE and MT– scans were resampled to 0.5 mm isometric resolution (ANTs' ResampleImage). Moreover, to facilitate template construction, participants' native space FSE scans were aligned to their template-space MPRAGE scans (antsRegistrationSyNQuick). Native space MT+ scans were aligned to resampled MT– scans to account for potential movement effects between scan acquisitions (antsRegistrationSyNQuick). After their alignment, MT– and MT+ scans were submitted to a common multimodal template construction, while FSE and MPRAGE scans each were used to generate a brainstem and whole-brain template, respectively.
Next (step 2), modality-specific group templates (MPRAGE, FSE, MT+ and MT–) were linearly and non-linearly coregistered (antsRegistration) to standard space (MNI-ICBM 152 linear, 0.5 mm). Specifically, templates with a sensitivity for catecholaminergic nuclei (FSE, MT+, MT–) were first standardized to the whole-brain MPRAGE template (using a coregistration mask). Next, the MPRAGE template was coregistered to MNI space and the transformations were applied to the other templates (FSE, MT+, MT–; antsApplyTransforms). To improve coregistration accuracy, whole-brain templates (MPRGAE, MT–, MNI) were skull stripped before alignment using the FMRIB Software Library.
Finally (step 3), all transformation matrices were concatenated and applied to individual participants' scans to bring them from native to MNI space in a single step (antsApplyTransforms).

Normalization template: MNI 152 linear 0.5 mm resolution

Noise and artifact removal: N4BiasFieldCorrection as implemented in Advanced Normalization Tools (ANTs; v. 2.3) antsMultivariateTemplateConstruction

Volume censoring: does not apply; we conducted structural analyses only

Statistical modeling & inference

Model type and settings: We used structural equation modeling (SEM) to evaluate inter- and intra-individual differences in catecholaminergic nuclei and their association with cognition . All models used full information maximum likelihood (FIML) estimation to account for missing values. The adequacy of the reported models was evaluated using $\chi2$-tests (i.e., an absolute fit index), as well as two frequently reported approximate fit indices: the root mean square error of approximation (RMSEA) and the comparative fit index (CFI). RMSEA values close to or below 0.06 and CFI values close to 0.95 or greater indicate good model fit. Unless otherwise noted, multi-group models were fit, comprising submodels for younger and older adults. For this, invariance across age groups was evaluated by a hierarchical series of likelihood-ratio tests, probing group differences in (1) factor loadings (weak invariance), (2) indicator intercepts (strong invariance), and (3) residual variances (strict invariance). In case of longitudinal models, the same criteria were applied to test invariance across time. After establishing adequate model fit and invariance, the significance of parameters of interest was evaluated using likelihood-ratio tests. That is, we created two nested models—in one, the parameter of interest was freely estimated, whereas in the other model it was fixed to zero. If a fixing the parameter of interest to zero resulted in a drop in model fit, as evaluated using a likelihood-ratio test comparing the two nested models, this indicated the significance of the parameter.

Effect(s) tested: Likelihood-ratio tests were used (see above) to test associations (correlation or regression paths) between latent neural and cognitive factors.

Specify type of analysis: ☐ Whole brain ☒ ROI-based ☐ Both

Anatomical location(s): Locus coeruleus ROI was determined based on a previously published consensus mask; see: https://doi.org/10.1016/j.neurobiolaging.2021.11.006
Substantia nigra-ventral tegmental area ROI was based on: https://doi.org/10.1073/pnas.1807983116

Statistic type for inference (See Eklund et al. 2016): We did not perform voxel-wise analyses but extracted overall-estimates of neuromodulatory integrity which were analyzed using SEM (see above).

Correction: We did not perform voxel-wise analyses but extracted overall-estimates of neuromodulatory integrity which were analyzed using SEM (see above).

Models & analysis

n/a	Involved in the study
☒	☐ Functional and/or effective connectivity
☒	☐ Graph analysis
☒	☐ Multivariate modeling or predictive analysis
